# Validation of the chronic disease score-infectious disease (CDS-ID) for the prediction of hospital-associated *clostridium difficile* infection (CDI) within a retrospective cohort

**DOI:** 10.1186/1471-2334-13-150

**Published:** 2013-03-26

**Authors:** Vanessa Stevens, Cathleen Concannon, Edwin van Wijngaarden, Jessina McGregor

**Affiliations:** 1 Pharmacotherapy Outcomes Research Center, University of Utah College of Pharmacy, 421 Wakara Way, Suite 208, Salt Lake City, UT, 84108, USA; 2 University of Rochester School of Medicine and Dentistry, Rochester, NY, USA; 3 Department of Pharmacy Practice, Oregon Health and Science University College of Pharmacy, Portland, OR, USA

**Keywords:** Comorbidity score, *Clostridium difficile* infection, Validation, Chronic disease score, Infectious disease (CDS-ID)

## Abstract

**Background:**

Aggregate comorbidity scores are useful for summarizing risk and confounder control in studies of hospital-associated infections. The Chronic Disease Score – Infectious Diseases (CDS-ID) was developed for this purpose, but it has not been validated for use in studies of *Clostridium difficile* Infection (CDI). The aim of this study was to assess the discrimination, calibration and potential for confounder control of CDS-ID compared to age alone or individual comorbid conditions.

**Methods:**

Secondary analysis of a retrospective cohort study of adult inpatients with 2 or more days of antibiotic exposure at a tertiary care facility during 2005. Logistic regression models were used to predict the development of CDI up to 60 days post-discharge. Model discrimination and calibration were assessed using the c-statistic and Hosmer-Lemeshow (HL) tests, respectively. C-statistics were compared using chi-square tests.

**Results:**

CDI developed in 185 out of 7,792 patients. The CDS-ID was a better standalone predictor of CDI than age (c-statistic 0.653 vs 0.609, *P*=0.04). The best discrimination was observed when CDS-ID and age were both used to predict CDI (c-statistic 0.680). All models had acceptable calibration (*P*>0.05).

**Conclusion:**

The CDS-ID is a valid tool for summarizing risk of CDI associated with comorbid conditions.

## 

What is New?

•CDS-ID was a good predictor of CDI (c=0.65)

•CDS-ID was a significantly better standalone predictor than age

•CDS-ID plus age resulted in the highest discrimination and the greatest degree of confounder control in our example

•There was no difference in models using CDS-ID compared to individual comorbidities

•CDS-ID is a valid tool to predict comorbidity-related risk in studies of CDI

### Background

The presence and severity of underlying comorbid conditions are important contributors to the risk of nosocomial infections such as *Clostridium difficile* Infection (CDI) [1-3]. The construct of comorbidity is complex because it represents information on a wide variety of disease states, underlying biological mechanisms, and a spectrum of disease severity. Many of these disease processes are also highly correlated, making the estimation of individual comorbidity-specific effects challenging. As a result, there has been considerable variability in the methods used to measure comorbidity in studies of risk factors for nosocomial CDI [1-6]. The most common method to control for potential confounding by underlying comorbidity is to include a set of binary variables indicating the presence or absence of each individual comorbid condition of interest, potentially resulting in inadequate statistical power because estimation of more variables requires more degrees of freedom. Compounding this problem is the fact that many studies of CDI are conducted in the setting of an outbreak and are limited by relatively small sample sizes [4,6], leading to the potential for overfitting of regression models and reducing power to detect true associations [7].

Aggregate comorbidity scores are most commonly used for risk stratification (e.g. prediction of an individual’s risk of disease based on the value of the score), confounder control, or some combination of both applications. Aggregate scores have the advantage of summarizing the risk of the outcome attributable to a particular set of covariates into one summary measure, thereby reducing the number of parameters to be estimated while still controlling for potential confounding effects. Well-known comorbidity scores such as the Charlson Index [8], Horn Index [9], and the Chronic Disease Score [10], while commonly used in studies of risk factors for CDI and other HAI [1,4,5,11] were developed specifically for the prediction of mortality and related outcomes and not the development of infection while in the hospital. Both the Charlson Index and the Chronic Disease Score were shown to perform sub-optimally for the prediction of nosocomial infection [12].

The Chronic Disease Score – Infectious Disease (CDS-ID) is an adaptation of the original Chronic Disease Score (CDS) for the assessment of comorbidity in relation to hospital-associated infection (HAI) based on medication orders written within 24 hours of hospital admission [10,13]. The CDS-ID was developed and validated to predict the acquisition of nosocomial vancomycin-resistant enterococci (VRE) or methicillin-resistant *Staphylococcus aureus* (MRSA) infections during hospitalization rather than mortality or health status [13]. Although developed specifically for VRE and MRSA, it is thought that this generalized score is potentially suitable for use in studies of other HAI because many hospital-associated infections share similar comorbidity-related risk factors [13]. However, it is well known that the performance of prognostic scores may vary significantly depending on the underlying characteristics of the population in which the score is used, particularly when the outcome of interest differs from that which the score was originally developed to predict [14,15]. Models using general comorbidity scores in some populations have been observed to predict disease no better than models including age alone, highlighting the need to verify performance of scores for new endpoints [15].

While CDS-ID has been used in at least one previous study of CDI and has been demonstrated to be significantly associated with the risk of CDI infection [16], the performance of the score for the prediction of CDI has not been fully evaluated. In addition, patient age tends to be associated with the burden of underlying illness and is usually readily available without additional data collection. It is unknown whether summary comorbidity scores provide sufficient improvement in outcome prediction to justify the effort and expense of collecting additional data. Therefore, the aim of the present study was to determine the discrimination and calibration of the CDS-ID for the prediction of CDI, to assess the relative importance of accounting for age either by itself or in concert with CDS-ID, and to assess the potential for confounding reduction with these measures, with specific application to the association between antibiotic exposure and the risk of CDI.

### Methods

#### Patient population

We used data from a previously-established cohort of adult patients receiving two or more days of antibiotics during an inpatient hospitalization occurring between 1 January and 31 December 2005 at the University of Rochester Medical Center (URMC) in Rochester, New York [16]. The primary aim of this study was to examine the association between cumulative, time-varying measures of antibiotic exposure and the risk of CDI. Eligibility criteria have been described elsewhere [16]. Briefly, patients were excluded if they had a history of CDI within 60 days prior to admission or if they developed CDI in the first 2 days of hospital stay, as infections developed during this time frame are considered to have been acquired in the community rather than in the hospital setting [17]. Individuals who were not at risk of the event because of a length of stay less than 2 days and those who received only short courses of antibiotics were excluded by not considering in the analysis any patients who received fewer than 2 days of antibiotic treatment. Patients were also excluded if they were admitted to a psychiatric ward. Hospital administrative databases, including pharmacy and billing records, were used to identify all patients who received two or more days of antibiotic exposure as inpatients during the study period. Information on patient demographics, units of stay during hospitalization, and dates of admission and discharge were also collected electronically. The secondary dataset used in this analysis contained only age, high risk antibiotic use, individual comorbidities and the CDS-ID score. Approval for this study was obtained from the URMC Research Subjects Review Board.

#### Outcome and predictor variables

For each qualifying hospitalization, information was obtained regarding the *International Classification of Diseases* – *9*^*th*^*Revision* (ICD-9) procedural and diagnostic codes associated with that hospital stay. Information was also available on the timing, dose, and route of administration of all medications received during each qualifying hospitalization. In the original study, patients were allowed to contribute more than one hospitalization to the analysis, and each hospitalization was followed for up to 60 days post-discharge for the development of CDI, defined as detection of *C*. *difficile* toxin in an unformed stool by enzyme immunoassay. Microbiology laboratory reports from all three laboratory systems that serve the hospitals and physicians’ offices in the community (URMC, Rochester General Hospital, and ACM clinical laboratory) were reviewed to monitor for post-discharge development of CDI. In order to avoid dependence among hospitalizations contributed by the same patient, we selected the first hospitalization contributed by each patient during the study period for the present analysis. Information was utilized regarding age, CDS-ID score, presence or absence of CDI at the end of the study period, and whether or not they were exposed to any antibiotics considered high risk for inducing CDI during the at-risk period. Agents were identified as high risk based on previous observations within this cohort, and include the following antibiotic classes: any cephalosporin or carbapenem, fluoroquinolone, intravenous vancomycin, or β-lactamase inhibitor combination [16].

For each patient, the CDS-ID score was calculated from information on medication orders written within the first 24 hours of hospital admission. Study investigators generated a list of all drugs received by all patients included in the study. Drugs were classified according to the main indication for which they are typically prescribed using Goodman and Gilman’s The Pharmacological Basis of Therapeutics [18] as well as input from clinical (medical and pharmacy) faculty members. Medications representing treatment for diabetes, peptic ulcer disease, respiratory illness, kidney disease, transplant, and cancer were identified, and patients were considered to have a particular comorbidity if they had been prescribed one of the agents used for its treatment. The presence of each comorbidity was weighted according to the regression coefficients identified in the development study [13]. Diabetes, peptic ulcer disease (PUD), respiratory illness, kidney disease, transplant, and cancer were worth 1.57, 1.83, 1.38, 3.13, 1.0, and 1.07 points, respectively. Regression weights were then summed across present conditions to provide a summary measure of the risk of CDI attributable to underlying illness. For example, a patient with diabetes and cancer would have a CDS-ID score of 2.64 (1.57 points for diabetes plus 1.07 points for cancer).

#### Data analysis

##### Descriptive analysis

The distributions of numeric variables (e.g. age and CDS-ID) were examined for normality using Kolmogorov-Smirnov test. Variables determined to be non-normally distributed (p<0.05) were described using the median and interquartile range, defined as the mathematical difference between the 75^th^ and 25^th^ percentile of the distribution. Clinical characteristics of patients with and without CDI were compared using Wilcoxon rank-sum tests for non-normally distributed numeric values, and Fisher’s exact or chi-square tests for categorical variables, as appropriate.

##### Model discrimination

In order to evaluate discriminatory ability, three logistic regression models were constructed. In the first and second models, the risk of CDI was estimated using CDS-ID and age, respectively, as the sole independent predictor variables. The third model expressed the risk of CDI as a function of both CDS-ID and age together. The assumption of a linear relationship between continuous predictor variables and the log of the odds was verified by visual inspection of plots of the log odds versus age and CDS-ID. For all models, the discriminatory ability was assessed using the c-statistic and 95% confidence intervals [19]. The c-statistic is a measure of the area under the receiver-operator characteristic (ROC) curve, which is a graphical representation of the sensitivity and specificity of outcome classification for all possible values of the predictor variable [20]. The c-statistic represents the probability that the value for a predictor variable will allow for accurate prediction of a patient with the outcome versus a patient without the outcome in a randomly chosen pair [20]. In practice, the c-statistic can be conceptualized as the percent of time that the model is able to accurately distinguish between cases and non-cases based on the values of predictors included in the model. For example, in a model with a c-statistic of 0.60 applied to 100 random case/non-case pairs, we would expect an accurate discrimination between the cases and non-cases in 60 of the 100 pairs, or 60%. Model discrimination was compared between models using chi-square tests for differences between c-statistics according to the methods for comparing the areas under two correlated ROC curves suggested by DeLong and colleagues [19]. The discrimination of models was also compared to a c-statistic of 0.5, or the equivalent of predicting outcome based on the flip of a coin, using chi-square tests.

##### Model calibration

Calibration, which measures the fit of a particular model to the data, was assessed using the Hosmer-Lemeshow (HL) goodness-of-fit test [21]. In this test, patients are divided into equal groups of predicted risk (typically deciles) for the outcome of interest. The distribution of observed cases is compared to the distribution of expected cases based on the specified model. The null hypothesis of the HL test is that there is no significant lack of model fit (e.g. no significant difference in the distribution of expected and observed cases); therefore a *P*-value less than 0.05 is associated with a significant lack of model fit. Increasing *P*-values (e.g., closer to 1) are generally associated with better model fit [22]. Because of the large sample size, the HL test is expected to be sensitive to small departures in the distributions of observed and expected cases. As such, both *P*-values and HL graphs were used to interpret model calibration.

##### Confounder control

The potential ability of the CDS-ID, when used as a covariate, to reduce the confounding effects of comorbidity was assessed using the relationship between high-risk antibiotic exposure and CDI as an illustrative example. Four separate logistic regression models were constructed. Unadjusted estimates of the association between high risk antibiotics and CDI were obtained from a model containing antibiotics as the only independent predictor. Additional models were constructed by adding additional adjustment variables: CDS-ID, Age, or both CDS-ID and age. Absolute and relative (%) changes to the main exposure odds ratio as compared to the unadjusted estimate were calculated. The performance of CDS-ID was compared to adjustment using the individual comorbid conditions comprising the score with respect to discriminatory ability, calibration, degree of confounding reduction, and precision of the main-effect estimates. Point estimates (e.g. odds ratios and c-statistics) and precision were similar across models (data not shown).

### Results

Of the 10,154 hospitalizations available for analysis, 7,792 first hospitalizations were chosen from unique patients. Among these patients, there were 185 episodes of CDI, resulting in an observed incidence of approximately 2.4%. A comparison of the baseline clinical characteristics of patients with and without CDI is shown in Table 1. Overall, patients with CDI were older on admission (median 69.9 versus 59.1 years, Wilcoxon rank-sum *P* < 0.01) and had a higher proportion of high risk antibiotic use (96.8% versus 90.3%, *P* < 0.01) as compared to patients that did not go on to develop CDI. Case patients also had a greater degree of comorbidity as measured by the CDS-ID score (median 2.83 versus 1.83 points, *P* < 0.01) and by the frequency of individual comorbid conditions included in the score, with the exception of organ transplant (7.6 versus 6.2, p=0.44).

**Table 1:** **Comparison of clinical characteristics of patients with and without *Clostridium difficile *Infection** (**CDI**)

**Characteristic**	**CDI n (%)**	**No CDI n (%)**	***P***
No. of Patients	185	7607	
Age, y, median (IQR)	69.6 (25.9)	59.1 (30.6)	<0.01
CDS-ID Score, median (IQR)	2.83 (1.57)	1.83 (3.21)	<0.01
Cancer	12 (6.5)	168 (2.2)	<0.01
Diabetes	65 (35.1)	1605 (21.1)	<0.01
Kidney Disease	19 (10.3)	289 (3.8)	<0.01
Peptic Ulcer Disease	143 (3.2)	4315 (96.8)	<0.01
Respiratory Illness	48 (26.0)	1517 (20.0)	0.04
Organ Transplant	14 (7.6)	470 (6.2)	0.44
Any High Risk Antibiotics ^a^	179 (96.8)	6868 (90.3)	<0.01

Abbreviations: IQR=Interquartile Range, CDI=Clostridium difficile infection, CDS-ID =Chronic Disease Score-Infectious Disease

^a^ Defined as any cephalosporin, carbapenem, fluoroquinolone, IV vancomycin, or β-lactamase inhibitor combination and derived from observed associations (ref).

The discriminatory abilities of models to predict CDI using age, CDS-ID, and both age and CDS-ID are shown in Table 2 The model employing age alone for the prediction of CDI risk resulted in a c-statistic of 0.609 (95% confidence interval (CI) 0.570, 0.649). Use of the CDS-ID score only represented a significant improvement over prediction using age only, yielding a c-statistic of 0.653 (95% CI 0.617, 0.689, chi-square *P*=0.04). Relative to the model containing age only, the addition of CDS-ID would result in accurate discrimination of an additional 7 out of every 100 random pairs (c-statistic 0.681;95% CI 0.646, 0.716). Figure 1 shows ROC curves constructed for models containing both CDS-ID and age and age alone. All models performed significantly better than random prediction (*P*<0.01 for all comparisons).

**Figure 1:**
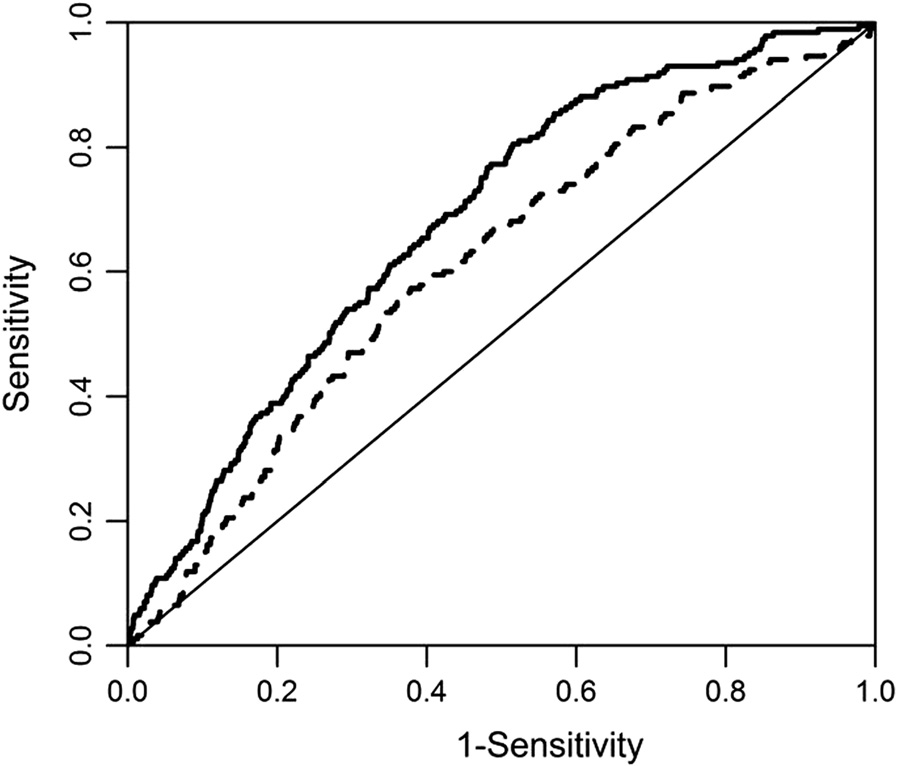
**ROC curves for models containing only age and CDS**-**ID with age as predictors of CDI.** (Dashed line – Age Only; Solid Line – CDS-ID plus Age; ROC - Receiver operator characteristics; CDI - *Clostridium difficile* Infection).

**Table 2:** **Comparison of discriminatory abilities of CDS**-**ID**, **age**, **and CDS**-**ID and age to predict *Clostridium difficile *Infection** (**CDI**)

**Model**	***c *Statistic**	**95% CI**	**Comparison to Age Alone Chi**-**square *P***
Age	0.609	0.570, 0.649	-
CDS-ID	0.653	0.617, 0.689	0.04
CDS-ID + Age	0.681	0.646, 0.716	<0.01

Abbreviations: CDI=Clostridium difficile infection, CDSID =Chronic Disease Score – Infectious Disease, CI=Confidence Interval.

The Hosmer-Lemeshow goodness of fit test indicated that model calibration was adequate for CDS-ID (*P*=0.07). The model employing age in the prediction of CDI had very good fit, with a *P*-value of 0.61. The distributions of observed and expected cases across categories of risk for CDS-ID and age are demonstrated in Figure 2, panels (a) and (b), respectively. The combination of CDS-ID and age resulted in a model calibration *P*-value of 0.07. Figure 3 shows the calibration plot for this model. Visual inspection of all three calibration plots revealed no obvious departure of observed from expected cases across risk groups for any of the models.

**Figure 2:**
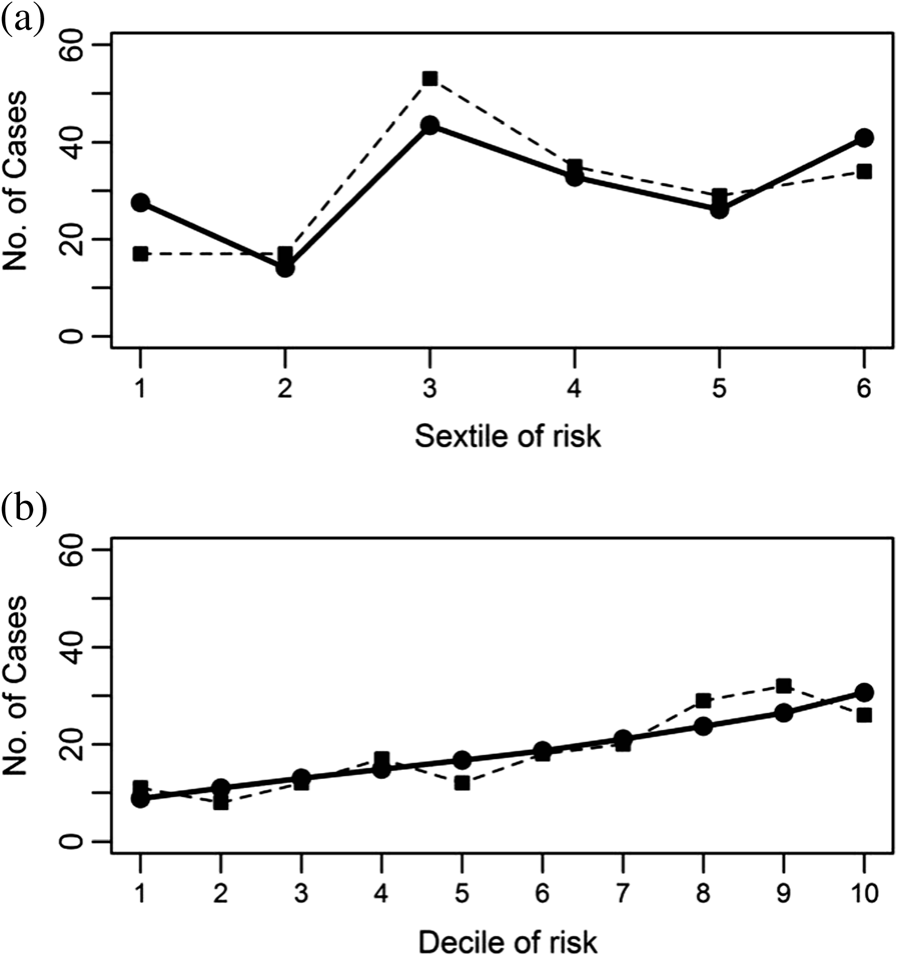
**Calibration plots of the CDS**-**ID** (**a**) **and age** (**b**) **as predictors of *Clostridium difficile *Infection.** (Solid line with circles – Expected Distribution of Cases; Dashed line with squares – Observed Distribution of Cases).

**Figure 3:**
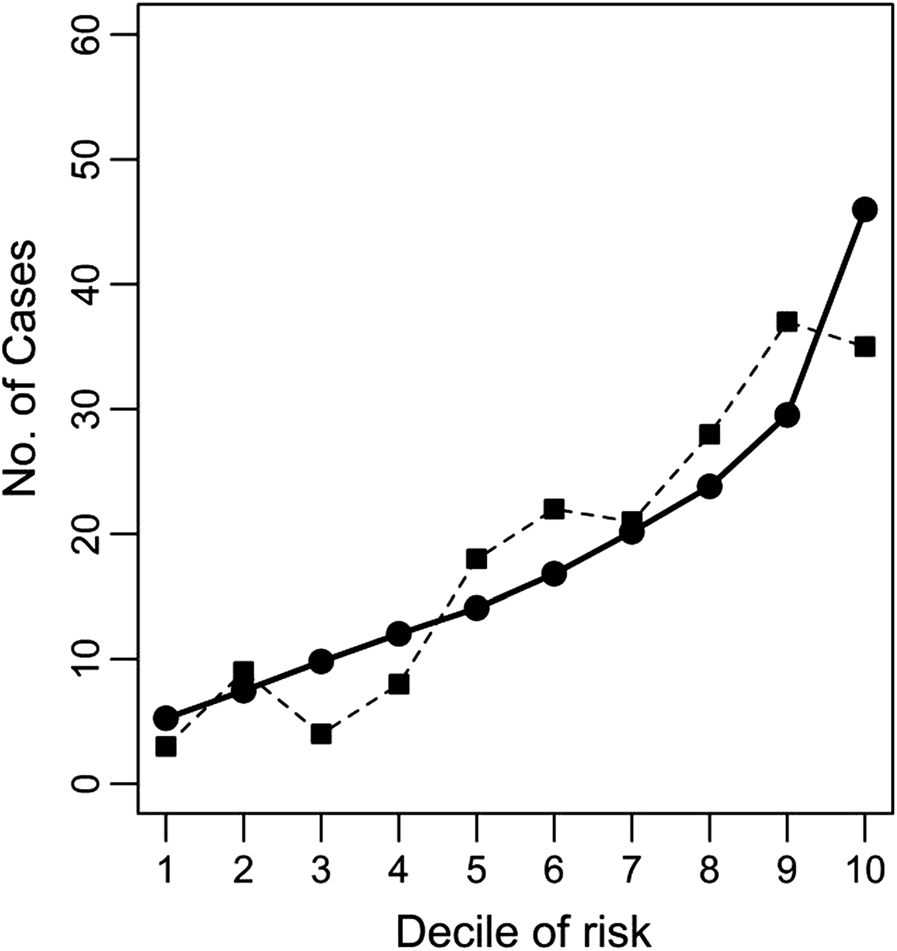
**Calibration plot of the CDS**-**ID plus age as predictors of *Clostridium difficile *Infection.** (Solid line with circles – Expected Distribution of Cases; Dashed line with squares – Observed Distribution of Cases).

The potential for confounding reduction by CDS-ID and age was assessed using the association between the use of high versus low risk antibiotics and the risk of CDI (Table 3). In univariate logistic regression, high risk antibiotic use was associated with a three-fold increase in risk of CDI as compared to the use of low risk antibiotics (OR 3.21, 95% CI: 1.42, 7.27, *P*=0.01). After adjustment for CDS-ID alone and age alone, the estimates for the main effect were reduced by 17% to 2.66 and by 20% to 2.56, respectively. Simultaneous adjustment for both CDS-ID and age resulted in the greatest reduction in the main effect risk estimate to 2.30, as well as the greatest precision, as measured by the width of the 95% CI (95% CI: 1.01, 5.23, *P*=0.04). The precision of the main effect estimates was similar for the models adjusted for CDS-ID only and age only (95% CI width 4.87 and 4.71, respectively).

**Table 3:** **Adjustment for Confounding Effects on the Association Between Antibiotic Exposure and the Risk of** (**CDI**)

**Model**	**Main Exposure Odds Ratio**	**95% CI**	**Change From Unadjusted Absolute (%)**	**c Statistic**	**95% CI**
High Risk Antibiotics	3.21	1.42, 7.27	-	0.532	0.519, 0.546
High Risk Antibiotics + CDS-ID	2.66	1.17, 6.04	−0.55 (17)	0.658	0.623, 0.694
High Risk Antibiotics + Age	2.56	1.13, 5.84	−0.65 (20)	0.621	0.582, 0.659
High Risk Antibiotics + CDS-ID + Age	2.30	1.01, 5.23	−0.91 (28)	0.686	0.651, 0.720

Adjustment for Confounding Effects of Comorbidity and Age on the Association Between High Risk Antibiotic Exposure and the Risk of *Clostridium difficile* Infection (CDI).

Abbreviations: OR= Odds Ratio, CI=Confidence Interval, CDI=Clostridium difficile infection, CDS-ID =Chronic Disease Score-Infectious Disease.

### Discussion

The presence of underlying comorbidities has consistently been implicated as a risk factor for the development of CDI [1,23,24]. Therefore, the control of potential confounding effects of comorbidities is important in studies of the etiology of CDI. Many such studies are conducted in the setting of outbreak investigations, resulting in small sample sizes and limited statistical power to adjust for multiple covariates. Even in larger studies, statistical power may be limited because of the relative infrequency with which CDI occurs [16,23,25]. In these situations, it may be impractical to adjust for all possible comorbid conditions as a set of binary predictor variables, and the use of an aggregate risk score may be preferable. Several scores have been developed to measure underlying comorbidity, but this has typically been done for specific outcomes, such as in-hospital mortality, that may have limited generalizability in studies where the outcome of interest is the development of HAI.

The CDS-ID score was developed as an adaptation of the original Chronic Disease Score for the prediction of VRE and MRSA infections [13]. Because of the overlap in risk factors for most nosocomial infections, the CDS-ID was considered generalizable to other infectious agents, but the performance of the score has not been verified specifically for use in studies of CDI. While the development of a new comorbidity score using study-specific weights would undoubtedly yield measures with greater discriminatory ability and model fit, the validation of a single measure across HAI outcomes may still be desirable for comparability across studies and for ease of application. This study represents the first formal evaluation of the performance of CDS-ID for the prediction of CDI. In addition, there is some question as to whether aggregate comorbidity scores contribute any additional information to the prediction of outcomes above that encompassed by age alone, which tends to be highly correlated with overall health status and degree of underlying comorbidity [15]. Given the added complexity of data collection and additional resources that may be required to compute aggregate risk scores, we also sought to verify that the CDS-ID represents an improvement over age adjustment alone in terms of either prediction, confounder control, or both for studies of CDI.

We observed that the use of CDS-ID represented a significant improvement over the discriminatory ability of random prediction alone, with a c-statistic of 0.653 (95% CI: 0.617, 0.689, *P*<.01). Similarly, age was also significantly better than random prediction (c-statistic 0.609, 95% CI: 0.570, 0.649, *P*<.01). While CDS-ID was a better predictor of CDI than age alone, the combination of these two predictor variables resulted in the best discriminatory ability, with a c-statistic of 0.681. In predictive models, discriminatory abilities above 0.70 are generally most desirable. However, comorbidities may influence the risk of CDI less directly than factors such as antibiotic use and colonization pressure (i.e. exposure to bacteria and spores) that are primary exposures in the causal pathway. Thus, predictive models that do not include these etiologic factors can be expected to have lower discriminatory abilities than models that account for variables with stronger causal associations. In addition, we found that CDS-ID performed as well or slightly better in the prediction of CDI than VRE (c-statistic 0.64) or MRSA (c-statistic 0.57) in the original development and validation samples [13].

Based on Hosmer-Lemeshow goodness-of-fit tests, none of the fitted models showed a significant lack of model fit. Models including CDS-ID had poorer fit than the model that included age alone (*P*=0.07 versus *P*=0.61, respectively). However, in HL tests, goodness-of-fit of the models is dependent on *P*-value, which in turn is influenced by sample size. Given the large number of subjects used in this study, small deviations of the distribution of observed cases from what is expected based on the model influence the assessment of model fit, even when these differences are not clinically relevant. Therefore, we also assessed model fit based on calibration plots. Visual inspection of the calibration plot for the CDS-ID only model showed overall good agreement of observed versus expected number of cases, with the largest discrepancy among individuals in the lowest sextile of risk. There was good agreement across deciles of risk for calibration plots for the other models as well. Overall, the models fit the data well.

In addition to their utility in risk stratification, aggregate comorbidity scores may be used for confounder control in studies with limited power due to small sample sizes. In order to assess the potential for confounding reduction, the association between the use of high risk antibiotics and the development of CDI was evaluated. Both age and comorbid conditions have been demonstrated as independent risk factors for CDI both in previous studies [5,26,27] and in our validation cohort [16,28]. Age has been shown to be related to appropriateness and quantity of antibiotic exposure [29,30], and it is reasonable to assume that patients with greater degree of underlying comorbidity, particularly those that are related to immune function, may be more likely to receive antibiotics compared to those with fewer comorbid conditions. On crude analysis, we found that high risk antibiotics were associated with a 3.2-fold increase in risk of CDI. After taking into account the effects of age, that estimate was reduced to 2.6. A similar reduction was observed after adjustment for CDS-ID. Co-adjustment for both age and CDS-ID resulted in a main effect estimate of 2.3, suggesting that there remains at least some variability in the outcome related to comorbidity that cannot be adequately explained by variation in age alone. Given the lack of gold standard for measuring true comorbidity, we were unable to assess to what extent CDS-ID was able to accurately measure these conditions. Similarly, we were unable to compare the performance of other common comorbidity scores in the prediction of CDI. However, our results indicate that adjustment for age alone is probably insufficient to account for confounding by underlying comorbidity, at least within the context of the example illustrated here. Interestingly, the dichotomous measure of high risk antibiotic use was near 0.50 for discrimination of CDI. It is possible that this is due to the lack of variability in the exposure, as more than 90% of the cohort received some high risk antibiotic during their hospitalization (data not shown). More detailed exposure assessments may result in better discriminatory abilities.

One of the primary limitations of the use of CDS-ID in etiologic studies of risk factors for CDI is the inclusion of PUD as one of the conditions. While still somewhat controversial, the evidence for an association between the use of acid suppressive agents, particularly proton pump inhibitors (PPI) and potentially histamine-2 receptor blockers (H2 blockers), is mounting [31]. Acid suppression is a cornerstone of treatment for peptic ulcer disease, and the presence of these agents would be incorporated as an indicator of PUD in CDS-ID. Investigators that wish to directly estimate the association between PPI or H2 blockers and the risk of CDI may not be able to do so because of the strong correlation between the score and individual medication use. In situations in which control for potential confounding by the use of acid suppressive agents is desired, the score alone may be sufficient, although this has not been investigated thoroughly. We also used detection of *C*. *difficile* toxins A and B by EIA in diarrheal stool as the method for case detection, which was the most common diagnostic test for CDI at the time of the study but since has been demonstrated to have sub-optimal sensitivity [32]. Application of CDS-ID in studies with alternative CDI testing algorithms such as polymerase chain reaction (PCR) or colonoscopy should not negatively impact the performance of the score, as the characteristics of these diagnostic tests (i.e. sensitivity and specificity) are unlikely to vary by CDS-ID score.

### Conclusion

Our analysis indicates that CDS-ID is a valid and useful tool for the measurement of underlying comorbid conditions in studies of the risk of nosocomial CDI. The use of CDS-ID resulted in a significantly improved discriminatory ability relative to age adjustment alone. Modeling the risk of CDI as a function of both age and CDS-ID resulted in the best discriminatory ability and the greatest degree of confounder control. Studies that examine risk factors for nosocomial CDI, particularly in the setting of limited sample size, may benefit from the use of CDS-ID to summarize risk of CDI associated with the degree of underlying comorbidity.

### Competing interests

All authors declare that they have no relevant conflicts of interest to report. This study was not supported by any grant funding.

### Authors’ contributions

VS, EvW, and JM were responsible for the Study concept and design. VS and EvW were responsible for data acquisition. VS and CC were responsible for data cleaning and statistical analysis. VS was responsible for manuscript preparation. VS, CC, EvW, and JM were responsible for data interpretation and manuscript review. All authors read and approve the final manuscript.

## Pre-publication history

The pre-publication history for this paper can be accessed here:

http://www.biomedcentral.com/1471-2334/13/150/prepub
